# Comprehensive Analysis of N6-Methyladenosine RNA Methylation Regulators in the Diagnosis and Subtype Classification of Acute Myocardial Infarction

**DOI:** 10.1155/2022/5173761

**Published:** 2022-08-24

**Authors:** Xianpei Wang, Ying Wu, Ruoyao Guo, Linwei Zhao, Juanjuan Yan, Chuanyu Gao

**Affiliations:** ^1^Department of Cardiology, People's Hospital of Zhengzhou University, Henan Provincial People's Hospital, Fuwai Central China Cardiovascular Hospital, Zhengzhou, Henan Province, China; ^2^Henan Provincial Key Lab for Control of Coronary Heart Disease, Zhengzhou University Central China Fuwai Hospital, Zhengzhou, China

## Abstract

Acute myocardial infarction (AMI) is still a huge danger to human health. Sensitive markers are necessary for the prediction of the risk of AMI and would be beneficial for managing the incidence rate. N6-methyladenosine (m6A) RNA methylation regulators have been confirmed to be involved in the development of various diseases. However, their function in AMI has not been fully elucidated. The purpose of this study was to determine the expression of m6A RNA methylation regulators in AMI as well as their possible functions and prognostic values. The GEO database was used to get the gene expression profiles of patients with and without AMI, and bioinformatics assays of genes with differently expressed expression were performed. We establish two separate m6A subtypes, and relationships between subtypes and immunity were studied. In this study, we identified IGF2BP1, FTO, RBM15, METTL3, YTHDC2, FMR1, and HNRNPA2B1 as the seven major m6A regulators. A nomogram model was developed and confirmed. The consensus clustering algorithm was conducted to categorize AMI patients into two m6A subtypes from the identified m6A regulators. Patients who have activated T-cell activities were found to be in clusterA; they may have a better prognosis as a result. Importantly, we found that patients with high METTL3 expressions had an increased level of Activated.CD4.T.cell and Type.2.T.helper.cell, while having a decreased level of CD56bright.natural.killer.cell, Macrophage, Monocyte, Natural.killer.cell, and Type.17.T.helper.cell. Overall, a diagnostic model of AMI was established based on the genes of IGF2BP1, FTO, RBM15, METTL3, YTHDC2, FMR1, and HNRNPA2B1. Our investigation of m6A subtypes may prove useful in the developments of therapy approaches for AMI.

## 1. Introduction

Heart failure (HF) has become a public health concern worldwide [1]. Although there has been an increase in the number of patients who are surviving HF thanks to recent advances in therapeutic strategies, the mortality rate for heart failure patients at five years remains within the 50 percent range, indicating that there is a need for additional focus on the prevention and treatment of heart failure [2, 3]. Acute myocardial infarction (AMI), which is a severe form of coronary heart disease, is one of the primary factors that result in the developments of heart failure [4, 5]. On reperfusion of acutely ischemic myocardium, myocardial damage and cardiomyocyte death occur due to a number of variables. This happens for a variety of reasons such as the opening of the mitochondrial permeability transition pore, ATP depletion, mitochondrial oxidative stress, and mitochondrial calcium overload. Medical therapy is the most effective treatment, but because there are no symptoms in the early stages, the prognosis is not good [6, 7]. Growing amounts of researches suggested that roughly 25 percent of those who survived an ST-segment elevation myocardial infarction went on to develop HF. Therefore, the early detection of AMI patients who are at a high risk of progressing to HF is essential for lowering the overall incidence of HF.

Growing studies have confirmed that epigenetic reprogramming plays a significant part in the developments of AMI [8, 9]. In recent years, RNA modification has been recognized as an important focus in epigenetic researches, and MODOMICS has roughly 172 distinct RNA modifications. An essential epigenetic change known as N6-methyladenosine (m6A) takes place when the adenosine base is methylated at the N6 position [10, 11]. In order for this modification to take place, the engagement of a large number of regulatory proteins is required. Significant functions are performed by regulators of m6A, the most common kind of RNA modification. These functions include destruction, translation, localization, transportation, and RNA processing [12]. The formation of m6A was modulated by three categories of proteins: readers (CBLL1, RBM15B, RBM15, ZC3H13, VIRMA, WTAP, METTL16, METTL14, and METTL3), 2 erasers (ALKBH5 and FTO), and 15 readers (IGF2BP1, ELAVL1, RBMX, IGFBP3, IGFBP2, IGFBP1, HNRNPA2B1, LRPPRC, FMR1, HNRNPC, YTHDF3, YTHDF2, YTHDF1, YTHDC2, and YTHDC1). Recent years have seen a proliferation of research that has shed light on the role that m6A-modified mRNAs play in the course of human cardiac disease [13, 14]. For example, ischemia-reperfusion injury (IRI) and hypoxia/reoxygenation (H/R) cardiomyocytes in mice have been shown to downregulate METTL3, whilst the overexpression of METTL3 has been shown to mitigate the IRI and H/R-induced cells' death [15]. Overall, evidence suggests that m6A was involved in the development of cardiovascular disease.

Infiltrating cells have a distinct geographical and temporal distribution and activity pattern, while at the same time, they are actively and continuously participating in cross-talk with one another and with other heart cells called cardiomyocytes [16, 17]. This results in the formation of a very complicated regulation pattern, which is an essential component in the normal recovery process of the heart following AMI [18, 19]. Inflammatory processes can also contribute to cardiac damage such as hypertrophy, fibrosis, and other forms, which can then lead to heart failure. The aseptic inflammation that results from AMI is characterized by the recruitment and activation of cells from both the innate and adaptive immune systems [20, 21]. Therefore, immunoregulatory treatment possesses a significant amount of potential for enhancing left ventricular remodeling and expediting cardiac healing following AMI [22, 23]. Discovering the time dynamics of immune cell buildup during AMI is crucial in order to locate the most effective immunoregulatory treatment [24, 25]. In previous research, immunohistochemical techniques were used to investigate immune cells [26]. These techniques relied on a single marker to recognize a particular subset of immune cells; however, the results cannot meet the clinical needs.

In this research, we investigated in a methodical manner the functions that m6A regulators play in the administration and classification of AMI. Our group developed a gene profile for the prediction of occurrences of AMI and found that treatment decisions based on this signature could be beneficial to patients. Additionally, we studied the association between two m6A subtypes and the infiltrating immune cell.

## 2. Materials and Methods

### 2.1. Microarray Data

The microarray data of GSE48060 were downloaded from the Gene Expression Omnibus database in NCBI, which were obtained based on the GPL570 platform of the Affymetrix Human Genome U133 Plus 2.0 Array. In order to develop an expression profiling method, a total of 52 blood samples from the periphery of the body were collected. These samples came from 31 patients who had suffered their first AMI within 48 hours of the event, and 21 samples came from controls who had no history of cardiac disease and had normal echocardiograms. Patients diagnosed with AMI had an average age of 53 (range: 38-65), while normal control subjects had an average age of 56 (range: 33-65). The 26 m6A regulators included 9 writers (CBLL1, RBM15B, RBM15, ZC3H13, VIRMA, WTAP, METTL16, METTL14, and METTL3), 2 erasers (ALKBH5 and FTO), and 15 readers (IGF2BP1, ELAVL1, RBMX, IGFBP3, IGFBP2, IGFBP1, HNRNPA2B1, LRPPRC, FMR1, HNRNPC, YTHDF3, YTHDF2, YTHDF1, YTHDC2, and YTHDC1).

#### 2.1.1. Construction of a Random Forest Model and Support Vector Machine Model

The “limma” R program was applied to examine the dysregulated m6A methylation regulators between AMI samples and normal samples [27]. The criterion for screening differential genes was set at *P* < 0.05, and the expressions of the genes were compared. Analyses of differential m6A methylation regulators were conducted with the use of Pearson correlation coefficients (*r* > 0.3, *P* < 0.05). In order to develop a training model for distinguishing AMI samples from normal samples, the SVM and RF approaches were chosen. In order to determine whether or not the model was accurate, we made use of tools such as the ROC curve, “boxplots of residual,” and “reverse cumulative distribution of residual.” The RF approach was utilized on the dysregulated m6A methylation regulators in order to screen AMI samples as opposed to normal samples, and the calculations were carried out by utilizing the “randomForest” library from the R programming language. Here, mtry and ntree were set to 3 and 500, respectively. In the 10-fold cross-validation process, the ntree that produced the fewest cross-validation errors was chosen as the best option. We used the best ntree to determine the relevance of differentially expressed m6A methylation regulators.

#### 2.1.2. Construction of a Nomogram Model

The “rms” package was applied to build a nomogram model that was predicated on the identified potential m6A regulators in order to make a prediction regarding the prevalence of AMI patients. The calibration curve was utilized in order to determine the degree to which our projected values were consistent with reality. Decision curve analysis (DCA) was performed, and a clinical impact curve was plotted to assess whether decisions based on the model were beneficial to the patient.

#### 2.1.3. Identification of Molecular Subtypes Based on the Significant m6A Regulators

The procedure known as consensus clustering is used to determine the number of each member's subgroup as well as check the clustering algorithm's reasonableness through the usage of resampling. Using the “ConsensusClusterPlus” package in R, a procedure known as consensus clustering was carried out in order to discover different m6A patterns.

#### 2.1.4. m6A Cluster Assays and Assessment of Immune Cell Infiltration

Based on m6A methylation regulators, consensus clustering was utilized to find m6A clusters based on m6A methylation regulators. This was accomplished with the help of the “ConsensusClusterPlus” R program. After that, ssGSEA methods were utilized to analyze the abundance of 23 immune cells in AMI in order to further examine the link, and a bar plot was built in order to illustrate the characteristics.

#### 2.1.5. Calculation of the m6A Score for Each Sample

Considering the individual differences, principal component analysis (PCA) algorithms were applied to calculate an m6A score for each sample to examine the m6A patterns. The PCA algorithm prioritizes the greatest group of closely connected (or unrelated) gene blocks in the set and deemphasizes the contributions of genes that are not tracked with other members of the set.

#### 2.1.6. Statistical Analysis

The entire statistical analysis was completed via R program 4.0.2. *T*-tests and Wilcoxon rank-sum tests were used in the analysis of quantitative variables, depending on the type of data. In order to determine the nature of the connection between m6A regulators, linear regression analyses were carried out. We used Kruskal-Wallis tests to determine whether or not there was a significant difference between the clusters. The *P* value less than 0.05 was considered to have statistical significance.

## 3. Results

### 3.1. Landscape of the 26 m6A Regulators in AMI

**Figure 1:**
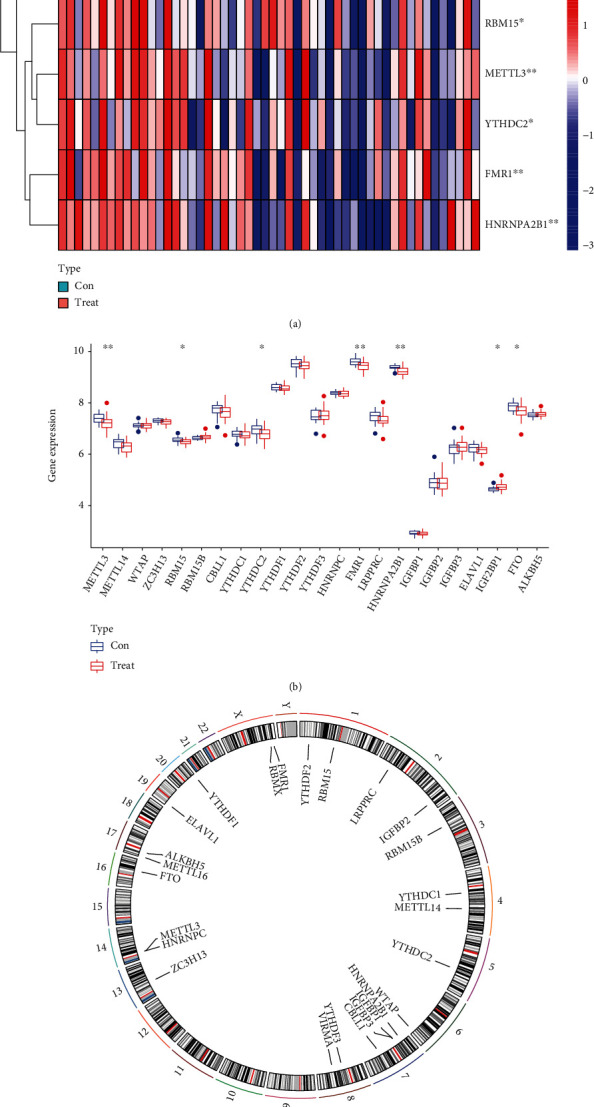
A comparison of the landscape of the m6A regulators that are dysregulated in AMI samples with normal samples. (a) A heat map comparing the levels of expression of the seven m6A regulators in samples from patients with AMI and normal patients. (b) Histogram comparing the dysregulated levels of the seven m6A regulators found in AMI samples with normal samples. (c) Locations on each chromosome of the 26 different m6A regulators.

Analysis of the dysregulated expressions of 26 m6A regulators between AMI samples and normal samples was performed using the “limma” package in the R programming language. IGF2BP1, FTO, RBM15, METTL3, YTHDC2, FMR1, and HNRNPA2B1 were the seven major m6A regulators that were identified and depicted by the use of a heat map (Figure 1(a)) and a histogram (Figure 1(b)). Using the “RCircos” software tool, the chromosomal locations of the 26 m6A regulators were mapped out and exhibited (Figure 1(c)).

### 3.2. Correlation between Writers and Erasers in AMI

**Figure 2:**
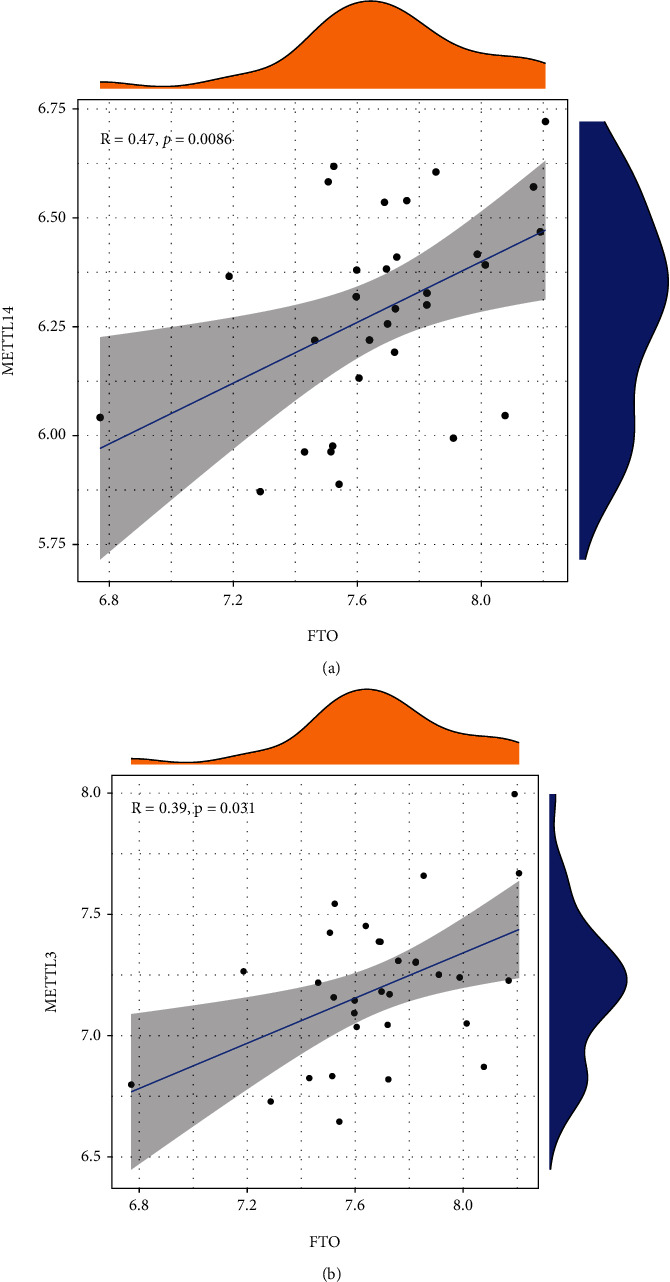
m6A regulators in AMI's correlation with one another. (a, b) Associations between erasers and writers in AMI.

In order to investigate whether AMI patients who have high writer gene expression levels also have low eraser gene expressions, we carried out linear regression analysis. These assays allowed us to investigate the degree to which writers and erasers are correlated. We discovered that there was a significant positive association between the levels of expression of METTL3 and METTL14 in AMI patients and FTO (Figures 2(a) and 2(b)).

### 3.3. Evaluation of the RF Model and SVM Model

**Figure 3:**
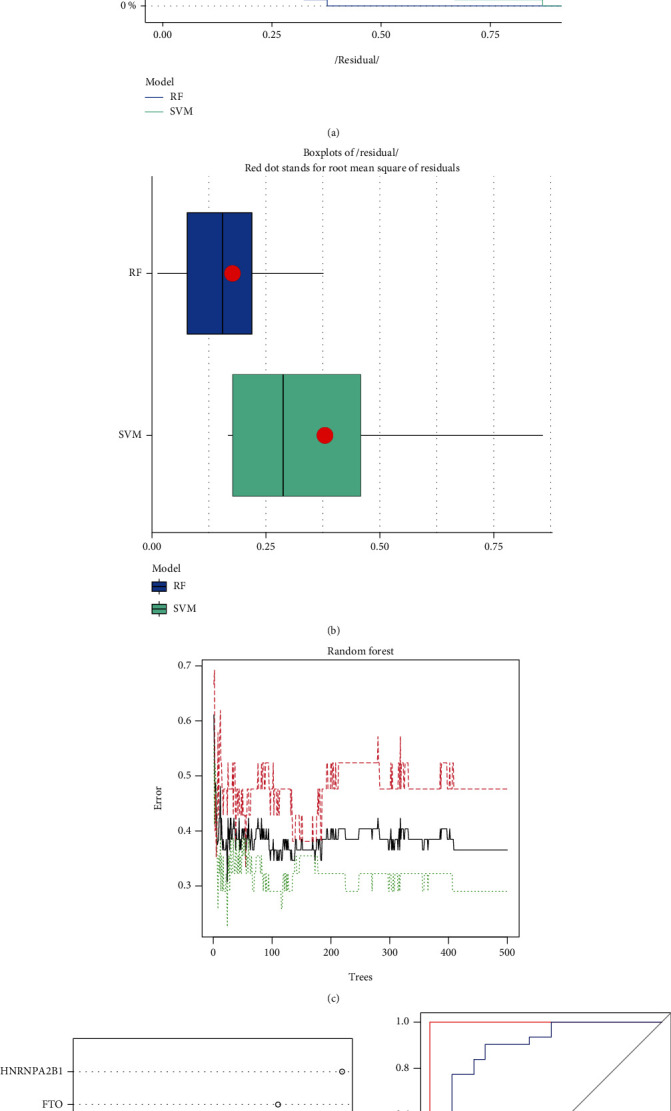
The development of both the RF model and the SVM model. (a) For the purpose of illustrating the residual distribution of the SVM and RF models, the reverse cumulative distribution of residual was presented. (b) Boxplots of residual were presented in order to illustrate the residual distribution of both models. (c) The correlation between the total number of decision trees and the error rate. (d) In light of the RF model, the significance of the seven m6A regulators is shown. (e) ROC curves showed that both the RF model and the SVM model were accurate.

Next, we developed an RF and SVM model in order to select the most effective m6A regulators from the DEGs described earlier in order to forecast the occurrence of AMI. The RF model with the fewest residuals was determined to be the best option (Figures 3(a) and 3(b)). Then, we decided to use 500 trees as the variables of the current model. This model showed a stable error possibility, so we went ahead and used it (Figure 3(c)). We also found that HNRNPA2B1 and FTO had a higher priority (Figure 3(d)). In addition, the ROC curves were constructed in order to evaluate the precision of these models, and the area under the curve (AUC) results revealed that the RF model has greater performance in comparison to the SVM model (Figure 3(e)).

### 3.4. Construction of the Nomogram Model

**Figure 4:**
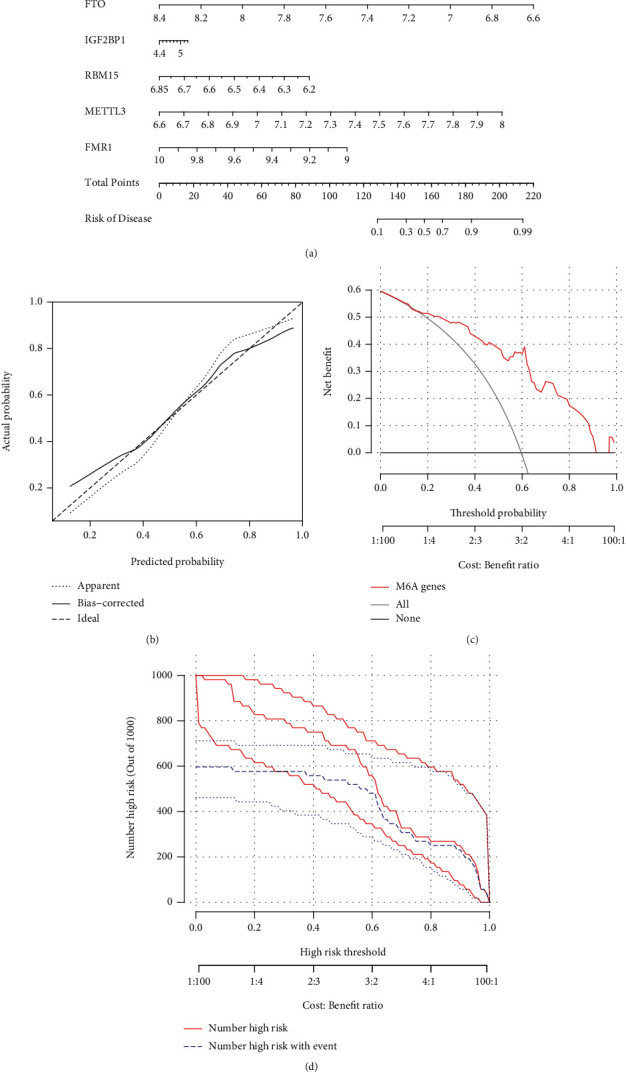
The diagnostic model of AMI that was developed using the random-forest method is presented in graphical form. (a) A nomogram model was constructed based on the seven candidates for m6A regulators. (b) Abilities of the nomogram model to make accurate predictions as shown by the calibration curve. (c) Patients suffering from AMI may benefit from decisions made using the nomogram model. (d) Evaluation of the clinical impact of the nomogram model using the clinical impact curve.

The “rms” package in R was used to develop a nomogram model that was by the use of the six candidates for the m6A regulator, and it was used for the prediction of the prevalence of AMI patients (Figure 4(a)). The predictivity of the new model was shown to be accurate through the use of calibration curves (Figure 4(b)). As shown in Figure 4(c), it may be deduced that judgments made by the use of the nomogram model may be beneficial to AMI patients. The clinical impact curve made it clear that the nomogram model possessed outstanding predictive ability (Figure 4(d)).

### 3.5. Two Distinct m6A Patterns Identified by Significant m6A Regulators

**Figure 5:**
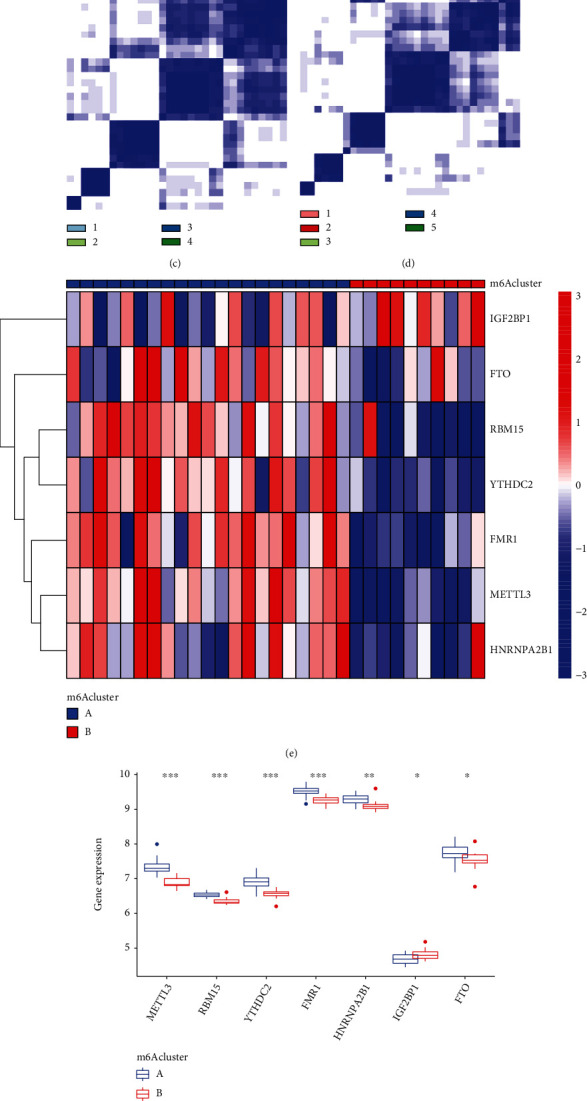
Clustering according to a consensus of the seven important RNA m6A regulators in AMI. (a–d) Consensus matrices of the seven significant m6A regulators for *k* = 2–5. (e) Heat map depicting the expression levels of the seven important m6A regulators in both clusterA and clusterB. (F) Histogram comparing the dysregulated levels of the seven important m6A regulators in clusterA and clusterB. (g) The PCA of the seven key m6A regulators reveals a striking dissimilarity in the transcriptomes of the two m6A patterns.

Using the “ConsensusClusterPlus” program in R software, the consensus clustering methods were applied to discover separate m6A patterns. This resulted in the identification of two distinct m6A patterns, which were denoted by the letters “clusterA” and “clusterB” (Figures 5(a)–5(d)). After that, a heat map and a histogram were constructed to highlight the expressions of the 10 major m6A regulators that can be found between the two clusters. IGF2BP1 revealed the reverse pattern, with higher expressions in clusterA than clusterB, whereas METTL3, RBM15, YTHDC2, FMR1, HNRNPA2B1, and FTO displayed lower expression levels (Figures 5(e) and 5(f)). According to the results of the PCA, the 10 important m6A regulators were able to completely differentiate between the two m6A patterns (Figure 5(g)).

**Figure 6:**
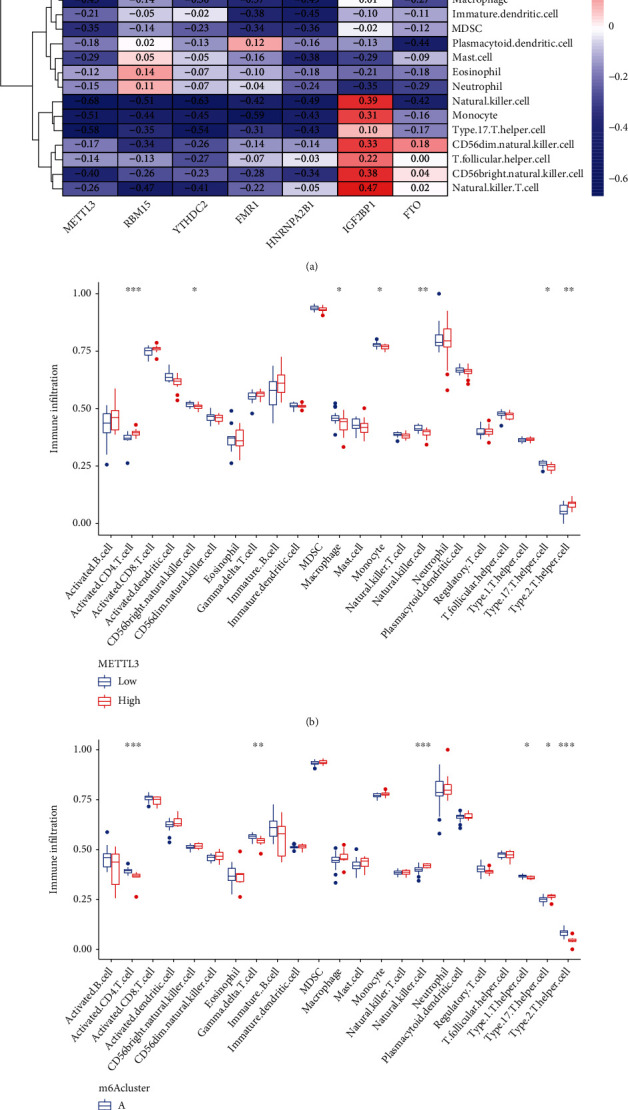
Comparison of the ssGSEA scores. (a) Infiltrating immune cells and the seven major m6A regulators have been found to have a correlation with one another. (b) Differences in the quantity of immune cells invading the tissue between groups with high and low levels of METTL3-associated protein expression. (c) Different patterns of immune cell infiltration were seen in clusterA and clusterB.

After that, we investigated the association between the seven important m6A regulators and the immune cells. Our research showed that METTL3 has positive relationships with a wide variety of immunological cells (Figure 6(a)). We investigated whether or not patients with high METTL3 expressions had a different type of immune cell infiltration than patients with low METTL3 expressions. We found that patients with high METTL3 expressions had an increased level of Activated.CD4.T.cell and Type.2.T.helper.cell, while having a decreased level of CD56bright.natural.killer.cell, Macrophage, Monocyte, Natural.killer.cell, and Type.17.T.helper.cell (Figure 6(b)). Finally, our group observed that clusterA was linked to Activated.CD4.T.cell, Gamma.delta.T.cell, and Type.17.T.helper.cell while clusterB was linked to Natural.killer.cell and Type.17.T.helper.cell (Figure 6(c)).

### 3.6. Evaluation of the m6A Gene Signature

**Figure 7:**
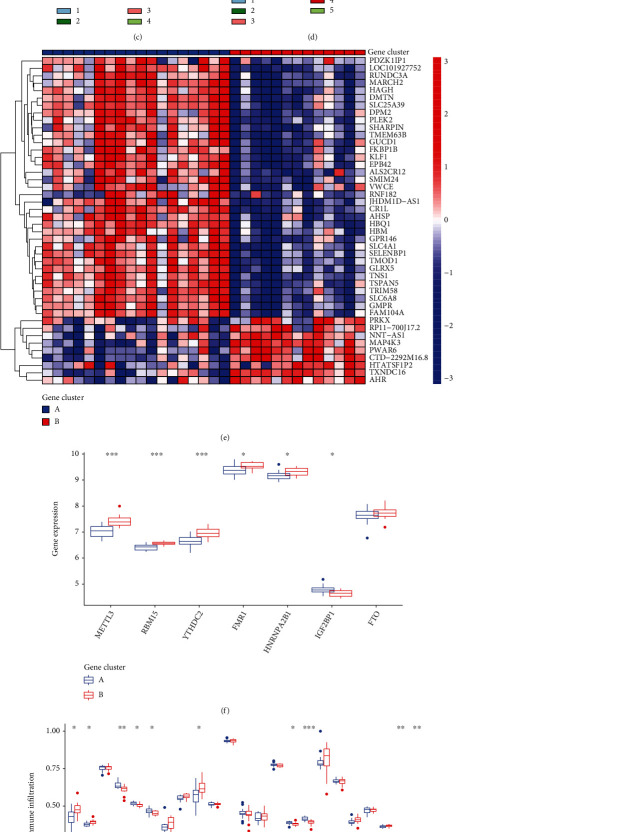
Consensus clustering of the 44 m6A-related DEGs in AMI. (a–d) Consensus matrices of the 44 m6A-related DEGs for *k* = 2–5. (e) Heat map depicting the expression of the 44 m6A-related DEGs that are members of gene clusterA and clusterB. (f) Histogram depicting the dysregulated levels of the 7 important m6A regulators between gene clusterB and gene clusterA. (g) A comparison of gene clusterA and gene clusterB reveals distinct patterns of immune cell infiltration. (h) Score differences on the m6A test between clusterB and clusterA. (i) Score differences on the m6A test comparing gene clusterB to gene clusterA.

**Figure 8:**
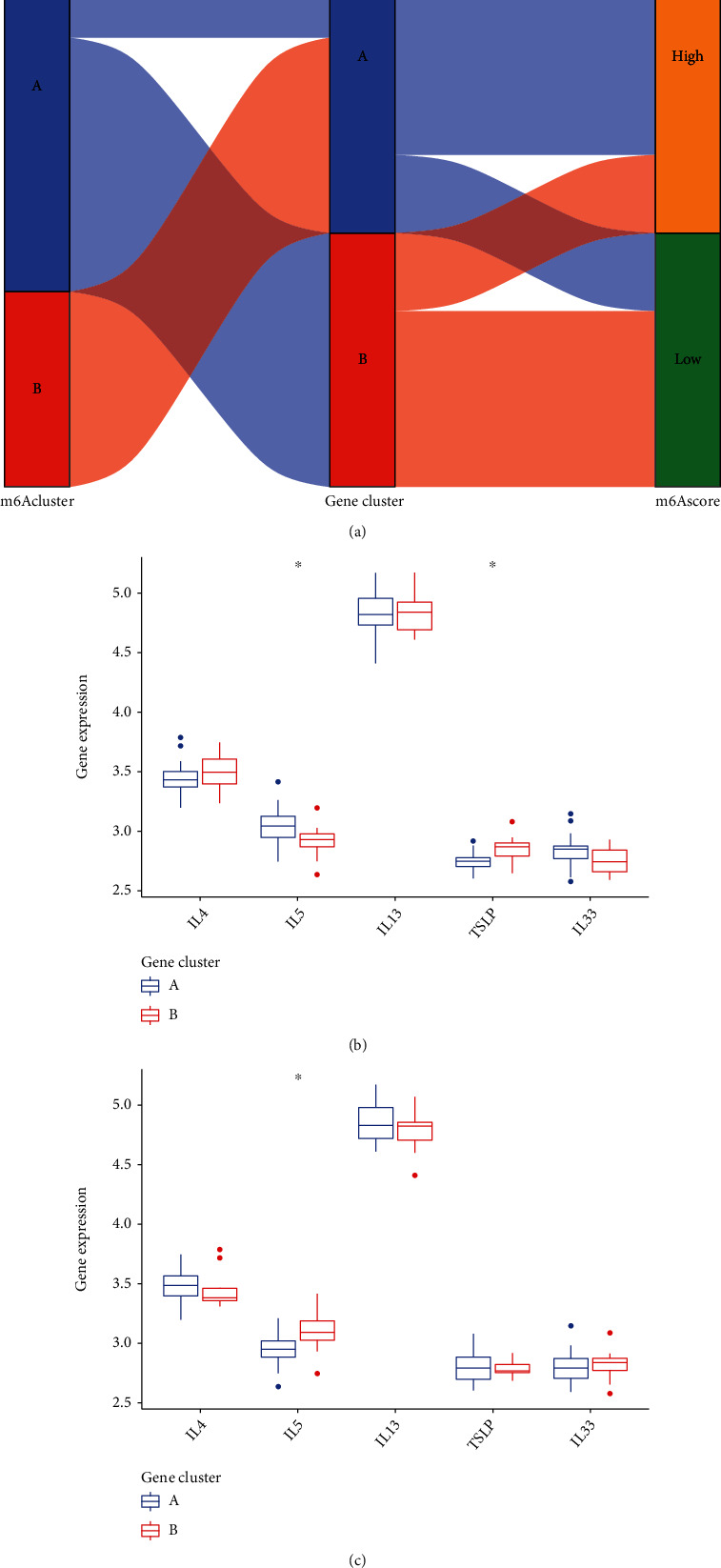
The potential ability of m6A patterns in screening AMI. (a) The link between m6A scores, m6A gene patterns, and m6A patterns is illustrated using the Sankey diagram. (b) The levels of five important factors were shown to be significantly different between clusterA and clusterB. (c) Comparison between gene clusterB and gene clusterA with regard to the dysregulated levels of five important factors.

The consensus clustering methods were applied to split the AMI patients into several genomic subtypes. These subtypes were determined by the number of DEGs. We discovered that there are two different m6A gene patterns in existence (Figures 7(a)–7(d)). The expressions of the 44 m6A-related DEGs in two groups were exhibited in Figure 7(e). After that, the dysregulation of the 18 m6A regulators and the immune cells between the various gene clusters was quite similar to that of the m6A subtypes (Figures 7(f) and 7(g)). The reasonableness of the clustering technique was shown by this result in its application to the subtype. Besides, Figures 7(h) and 7(i) illustrate the association that existed between m6A scores, m6A gene clusters, and m6A clusters. A Sankey diagram was used to illustrate the connection between the three groups (Figure 8(a)).

### 3.7. Relationship between m6A Subtypes and Cytokines

In order to get more detailed research on the connection between m6A patterns and AMI, we looked into the relationship that exists between m6A patterns and IL-13, IL-5, IL-4, IL-33, and TSLP. According to the findings, the expressions of TSLP were found to be greater in gene clusterB than those found in gene clusterA, but the expressions of IL-5 were found to be smaller in gene clusterB (Figure 8(b)). In addition, the findings demonstrated that the levels of expression of IL-5 in clusterB were significantly higher than those seen in clusterA (Figure 8(c)).

## 4. Discussion

Despite significant advances made in early detection and treatment over the course of the last decade, AMI continues to be a primary cause of death and disabilities [28]. As a direct consequence of this, the clinical outlook for patients diagnosed with AMI is not favorable [29]. Patients who have AMI frequently do not get the chances to benefit from the specific treatments because there is not yet a reliable method for diagnosing the condition early on, which results in poor outcomes [30–32]. Growing studies have confirmed that immune cell infiltration was involved in the onset and progression of AMI. Thus, many investigators are actively looking for potential markers and investigating the components of immune cell infiltration of AMI, both of which have the potential to have a significantly beneficial impact on the long-term survivals of patients with AMI. In recent years, many studies have reported the potential of mRNAs used as potentially useful indicators in the field of cardiovascular illness, specifically AMI. More and more data suggested that m6A regulators exhibited a regulatory effect on a wide variety of biological activities. Despite this, very little is known about the role that m6A regulators play in AMI. Our research was aimed at studying the functions of m6A regulators in AMI as the overarching goal.

In order to develop a model for the analysis and classification of AMI, the genes associated to m6A were subjected to a random-forest analysis. Both the calibration and the DCA graphs demonstrated that the model was a very excellent fit for the data. The model demonstrated that methylation was a significant molecular alteration that contributes to the progression of AMI. Under the model, the 6 most important genes were FTO, HNRNPA2B1, IGF2BP1, RBM15, METTL3, FMR1, and YTHDC2. We also ranked the 7 DEGs according to their relative gene relevance, and the results of this revealed that HNRNPA2B1 and FTO had a higher priority. In addition, we discovered that a reduction in the expression of FTO and an increase in demethylation activity led to an increase in the amount of m6A in RNA. This, in turn, produced a reduction in the contraction of hypoxic cardiomyocytes, which resulted in heart failure. It was found that HNRNPA2B1 directly binds a group of nuclear transcripts and modifies their alternative splicing in a manner that is analogous to how METTL3, the “writer” of the m6A gene, does it [33, 34]. Furthermore, HNRNPA2B1 was found to increase primary miRNA processing, thereby phenocopying the effects of METTL3 [33]. The potential function of the other important genes in various diseases was also reported.

The clustering technique allowed for the successful separation of 31 patients diagnosed with AMI into two distinct categories. Both the expression of genes related to m6A as well as the infiltration of immune cells were distinctly diverse between the two subtypes. It is important to note that the m6A-related genes were, on average, expressed at a higher level in Type A AMI compared to Type B AMI [35]. Additionally, immune cell infiltration was more common in Type A AMI. It has also been investigated whether or if there is a connection between modifications to m6A and the invasion of immune cells in malignancies. In addition, we selected METTL3 for further immunological testing because of its potency. We investigated the possible difference in infiltration immune cells between patients whose METTL3 expression levels were high or low. We found that patients with high METTL3 expression has an increased level of Activated.CD4.T.cell and Type.2.T.helper.cell, while having a decreased level of CD56bright.natural.killer.cell, Macrophage, Monocyte, Natural.killer.cell, and Type.17.T.helper.cell. Finally, we investigated the differences in immune cell infiltration that the two m6A patterns exhibited from one another. We found that clusterA was linked to Activated.CD4.T.cell, Gamma.delta.T.cell, and Type.17.T.helper.cell while clusterB was linked to Natural.killer.cell and Type.17.T.helper.cell. A previous study has reported that the degree of infiltration of B cells, CD4+ T cells, and CD8+ T cells was shown to have a negative correlation with the risk score, but the level of neutrophil, macrophage, and dendritic cell infiltration was found to have a positive correlation with the risk score.

The majority of researchers, as of right now, are of the opinion that a dysfunction in the Th cell subsets may be a significant connection in the process of the pathogenesis of AMI [36, 37]. When an external allergen enters the body, it is first identified and absorbed by antigen-presenting cells. The differentiation of Th cells regulated by IL-33 and TSLP was observed to result in an immune imbalance. In order to get more detailed research of the connection between m6A patterns and AMI, we looked into the relationship that exists between m6A patterns and IL-13, IL-5, IL-4, IL-33, and TSLP. We observed that the expressions of TSLP were increased in gene clusterB than in gene clusterA. In addition, we found that the expressions of IL-5 were distinctly increased in clusterB.

Our study contains a few caveats that need to be taken into consideration. First, this m6A-related gene signature was only verified in a select group of individuals. To further investigate and verify the results of this research, we recommend conducting a randomized controlled trial with a larger number of participants. Second, it was necessary to conduct additional experimental validations in order to investigate the molecular mechanism underlying m6A alterations in the progression of AMI.

## 5. Conclusions

In total, the current study selected five candidate m6A regulators (IGF2BP1, FTO, RBM15, METTL3, YTHDC2, FMR1, and HNRNPA2B1) and established a nomogram model that accurately predicts the prevalence of AMI. Collectively, our research may be able to provide further details for a better understanding of the functions that m6A plays in AMI, which may lead to a fresh perspective on the identification of biomarkers for the treatment and diagnosis of AMI.

## Data Availability

The data used to support the findings of this study are available from the corresponding authors upon request.
